# Hyperplastic candidosis on the palate developed as a “kissing” lesion from median rhomboid glossitis

**DOI:** 10.1590/S1808-86942010000100023

**Published:** 2015-10-17

**Authors:** Vivian Cunha Galletta, Márcia S Campos, Silvio K Hirota, Dante A Migliari

**Affiliations:** 1 Professional master's degree in lasers in dentistry, LELO-IPEN, Sao Paulo University (USP). Doctoral student in buccal diagnosis, Stomatology Department, Dentistry School, USP; 2 Master's degree in biological science, UNIVAP. Doctoral student in oral pathology, Stomatology Department, Dentistry School, USP; 3 Master's degree in buccal diagnosis, Stomatology Department, Dentistry School, USP. Doctoral student in buccal diagnosis, Stomatology Department, Dentistry School, USP; 4 Senior associate professor, Stomatology Department, Dentistry School, USP. Associate professor, Buccal Semiology Discipline, Stomatology Department, Dentistry School, USP. Dentistry School, USP

**Keywords:** oral candidiasis, oral diagnosis, tongue diseases

## INTRODUCTION

Chronic hyperplastic candidosis (CHC) affects predominantly adult males in the jugal mucosa commissure, and less frequently on the lateral tongue and palate. Smoking and occlusal friction are the most common local factors associated with CHC.^1^ This article reports a case of CHC on the palate resulting from candidosis associated with median rhomboid glossitis, characterizing an uncommon event known as specular lesion.

## CASE REPORT

**Figure 1:**
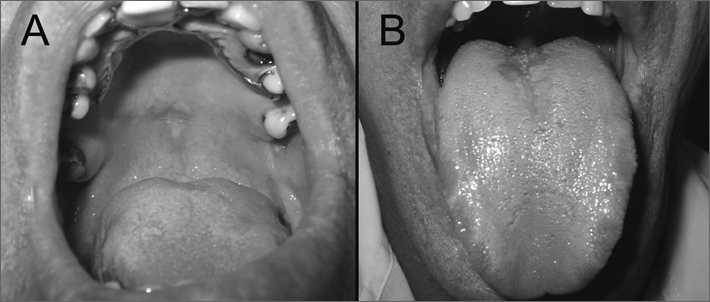
Initial presentation showing a white plaque on the palate mucosa (A) and the opposing lesion on the dorsum of the tongue (B). Note the similar contour of both lesions.

A male white patient aged 41 years was referred to the Stomatology Unit with a histopathological diagnosis of hyperplastic candidosis with moderate dysplasia on the palate. The clinical examination showed a white plaque measuring about 2 cm in diameter, with an erythematous halo, not removable by scraping (Fig. 1a). Also there was an erythematous atrophic area over which was a white plaque shaped similarly to the palatal lesion, on the dorsum of the tongue, which suggested candidosis associated with median rhomboid glossitis (Fig. 1b). Cytology and PAS staining confirmed the presence of Candida hyphae.

The patient had no history of systemic diseases and reported not using systemic or local medication. Laboratory exams did not reveal anemia, diabetes, or HIV infection. The patient used a partial removable dental appliance that did not make contact with the palatal lesion. The patient smoked but did not consume alcohol chronically.

Treatment with oral miconazole 20 mg gel, TID, during four weeks, resolved significantly both lesions. Cytology of both areas after treatment showed that Candida was absent; a biopsy of the residual lesion revealed hyperkeratosis without dysplasia.

## DISCUSSION

CHC is a separate variant of Candida infection because it generally presents epithelial dysplasia. CHD may present clinically as small nodular lesions to homogeneous white plaques not removable with scraping. The treatment of CHC is done with antifungal drugs followed by periodic clinical reassessments of the patient, as this type of candidosis has been associated with malignant transformation.^1^,^2^

There are few reports of Candida infection in the palate associated with median rhomboid glossitis, although dissemination of this infection would be expected due to permanent contact between the tongue and the palate mucosa.^3^ Non-dissemination of infection is probably due to the resistance of the palate mucosa to adhesion of hyphae, and the lubricant and cleaning action of saliva.

In this case report, the single local etiological factor found for CHC on the palate was candidosis on the dorsum of the tongue associated with median rhomboid glossitis. Although smoking was investigated as a possible predisposing factor, we believe that this habit did not cause the fungal infection, as the patient continued smoking during 16 follow-up months and the lesions did not relapse.^4^

## FINAL COMMENTS

The histological diagnosis of CHC is difficult because its features are similar to those of leukoplakia infected with Candida. It is still controversial whether Candida has a primary role in the etiology of CHC or if it invades a preexisting keratotic lesion. Full remission after antimycotic therapy confirms the diagnosis of CHC, although residual lesions - whitish mucosa of varying intensity - have been described, especially on the hard palate.^5^ The palate lesion regressed significantly after antimycotic therapy in this case; the epithelium became thinner and dysplasia or Candida hyphae became absent.

Local factors generally cause palate candidosis (e.g., maxillary appliances, poor oral hygiene or inhaling steroids), but systemic conditions may help the development of this condition.^1^ Having excluded these common etiological factors, the presence of median rhomboid glossitis associated with Candida should be investigated as a possible cause of palate candidosis, which characterized the so called “specular” lesion.^6^
